# Modality selective roles of pro-nociceptive spinal 5-HT_2A_ and 5-HT_3_ receptors in normal and neuropathic states

**DOI:** 10.1016/j.neuropharm.2018.09.028

**Published:** 2018-12

**Authors:** Ryan Patel, Anthony H. Dickenson

**Affiliations:** University College London, Gower Street, Department of Neuroscience, Physiology and Pharmacology, London, WC1E 6BT, UK

**Keywords:** In vivo electrophysiology, Ventral posterolateral thalamus, Spinal nerve ligation, Neuropathic pain, Descending facilitation, Serotonergic pain modulation, Ondansetron, Ketanserin, 5-HT_2A_, 5-HT_3_, CPM, conditioned pain modulation, R, receptor, RM, repeated measures, SNL, spinal nerve ligated, STT-VP-S_1/2_, spinothalamic tract-ventral posterior-somatosensory cortex, VPL, ventral posterolateral nucleus, WDR, wide dynamic range, 5-HT, 5-hydroxytryptamine

## Abstract

Descending brainstem control of spinal nociceptive processing permits a dynamic and adaptive modulation of ascending sensory information. Chronic pain states are frequently associated with enhanced descending excitatory drive mediated predominantly through serotonergic neurones in the rostral ventromedial medulla. In this study, we examine the roles of spinal 5-HT_2A_ and 5-HT_3_ receptors in modulating ascending sensory output in normal and neuropathic states. *In vivo* electrophysiology was performed in anaesthetised spinal nerve ligated (SNL) and sham-operated rats to record from wide dynamic range neurones in the ventral posterolateral thalamus. In sham rats, block of spinal 5-HT_3_Rs with ondansetron revealed tonic facilitation of noxious punctate mechanical stimulation, whereas blocking 5-HT_2A_Rs with ketanserin had minimal effect on neuronal responses to evoked stimuli. The inhibitory profiles of both drugs were altered in SNL rats; ondansetron additionally inhibited neuronal responses to lower intensity punctate mechanical stimuli and noxious heat evoked responses, whereas ketanserin inhibited innocuous and noxious evaporative cooling evoked responses. Neither drug had any effect on dynamic brush evoked responses nor on spontaneous firing rates in both sham and SNL rats. These data identify novel modality and intensity selective facilitatory roles of spinal 5-HT_2A_ and 5-HT_3_ receptors on sensory neuronal processing within the spinothalamic-somatosensory cortical pathway.

## Highlights

• Spinal 5-HT_3_Rs facilitate noxious mechanical sensory transmission in sham rats.
• Spinal 5-HT_2A_R block reveals minimal tonic activity in sham rats.
• Enhanced descending facilitation of evoked but not spontaneous neuronal activity in SNL rats.
• Cold and heat transmission facilitated via spinal 5-HT_2A_ and 5-HT_3_Rs respectively in SNL rats.

## Graphical abstract

**Figure:**
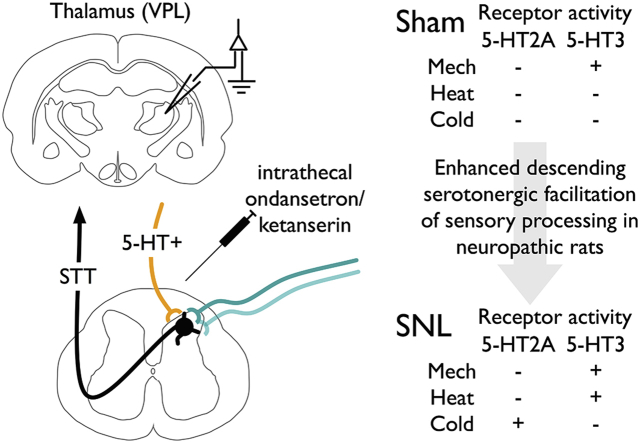

## 1 Introduction

Brainstem nuclei and higher brain centres can exert powerful modulation of nociceptive processing at the spinal level. This bi-directional control serves to amplify or suppress sensory transmission depending on context, expectation and emotional state. This is elegantly demonstrated during placebo analgesia, which is in part dependent on descending opioidergic pathways (Eippert et al., 2009). In addition, a recently identified bulbospinal projection is implicated in acute stress-induced hypoalgesia and chronic stress-induced hypersensitivity (Francois et al., 2017). Descending modulation is largely orchestrated via the periaqueductal grey (PAG), locus coeruleus and rostral ventromedial medulla (RVM) (Ossipov et al., 2014), although cortical regions such as the cingulate can exert direct facilitatory influences on spinal excitability (Chen et al., 2018), or indirectly via cortical-sub-cortical networks engaging descending brainstem pathways (Tan et al., 2017).

It is clear parallel inhibitory and excitatory pathways originating from the RVM exist (Zhuo and Gebhart, 1992). Neurones within the RVM display distinct firing patterns in response to noxious somatic stimulation; quiescent ON-cells begin firing and are considered to mediate descending facilitation, whereas tonically active OFF-cells abruptly cease firing and are considered to exert inhibitory influences (Fields et al., 1983; Heinricher et al., 1989), and a proportion of these sub-populations appear to be serotonergic (Gau et al., 2013; Mason, 1997). Numerous lines of evidence indicate facilitatory influences predominate. Selective optogenetic activation of medullary serotonergic neurones decreases nociceptive response thresholds (Cai et al., 2014). Lidocaine block of the RVM or depletion of spinal 5-HT decreases spinal neuronal excitability consistent with tonic facilitatory activity in normal states (Bee and Dickenson, 2007; Rahman et al., 2006). The ablation of NK1+ projection neurones in the dorsal horn with a saporin-substance P conjugate also suppresses deep dorsal horn neuronal excitability (Suzuki et al., 2002), revealing the parabrachial-RVM pathway as the efferent arm of a spino-bulbo-spinal circuit acting as a positive feedback loop facilitating spinal neuronal responses during noxious stimulation (Roeder et al., 2016; Suzuki et al., 2002).

Neuropathy and chronic pain states can be associated with increased descending facilitation; this time-dependent change in enhanced excitatory drive, and the failure to recruit inhibitory pathways (De Felice et al., 2011), promotes the transition from acute to chronic pain states and sustains persistent long-term pain (Burgess et al., 2002; Okubo et al., 2013; Wang et al., 2013; Wei et al., 2010; You et al., 2010). However, the precise roles of spinal 5-HTRs in different states have been difficult to characterise due to their complex dual pro- and anti-nociceptive functions, and the selectivity of available antagonists. However, we and others have reported key roles of the 5-HT_3_R in descending facilitations in a number of pain models (Bannister and Dickenson, 2016). In this study, we examine whether spinal 5-HT_2A_ and 5-HT_3_ receptors have intensity-dependent and modality-selective roles in modulating ascending sensory output, and how these functions are altered in a neuropathic state. We blocked spinal 5-HT_2A_Rs with ketanserin and 5-HT_3_Rs with ondansetron, and determined the effects on sensory neuronal coding in the ventral posterolateral thalamus (VPL). The STT-VP-S_1/2_ pathway is a key sensory-discriminative relay, and wide dynamic range (WDR) neurones in the rat VPL exhibit intensity-dependent coding across sensory modalities (Patel and Dickenson, 2016). Spinal WDR neurones code sensory inputs in a similar manner to human psychophysics, and can provide insight into sensory processing in normal and sensitised states in rodent models (Coghill et al., 1993; O'Neill et al., 2015; Sikandar et al., 2013b). Furthermore, these neuronal characterisations permit study of drug effects on stimulus intensities and modalities not amenable to behavioural testing in animals.

## 2 Methods

### 2.1 Animals

Sham or spinal nerve ligated (14–19 days post-surgery) male Sprague-Dawley rats (250–300 g) were used for electrophysiological experiments (Biological Services, University College London, UK). Animals were group housed (maximum of 4) on a conventional 12 h: 12 h light-dark cycle; food and water were available *ad libitum.* Temperature (20–22 °C) and humidity (55–65%) of holding rooms were closely regulated. All procedures described here were approved by the UK Home Office, adhered to the Animals (Scientific Procedures) Act 1986, and were designed in accordance with ethics guidelines outlined by the International Association for the Study of Pain (Zimmermann, 1983).

### 2.2 Spinal nerve ligation (SNL) surgery

SNL surgery was performed as previously described (Ho Kim and Mo Chung, 1992). Rats (120–130 g) were maintained under 2% v/v isoflurane anaesthesia delivered in a 3:2 ratio of nitrous oxide and oxygen. Under aseptic conditions a paraspinal incision was made and the tail muscle excised. Part of the L5 transverse process was removed to expose the left L5 and L6 spinal nerves, which were then isolated with a glass nerve hook (Ski-Ry, London, UK) and ligated with a non-absorbable 6-0 braided silk thread proximal to the formation of the sciatic nerve. The surrounding skin and muscle was closed with absorbable 3-0 sutures. Sham surgery was performed in an identical manner omitting the nerve hook/ligation step. All rats groomed normally and gained weight in the following days post-surgery. Establishment of the model was confirmed by determining mechanical withdrawal thresholds as previously described (Patel et al., 2014) (data not shown).

### 2.3 In vivo electrophysiology

Electrophysiology was performed as previously described (Patel et al., 2018b). Rats were initially anaesthetised with 3.5% v/v isoflurane delivered in 3:2 ratio of nitrous oxide and oxygen. Once areflexic, a tracheotomy was performed and rats were subsequently maintained on 1.5% v/v isoflurane for the remainder of the experiment (approximately 4–6 h, core body temperature was regulated with a homeothermic blanket throughout and respiratory rate was visually monitored). Rats were secured in a stereotaxic frame, and after the was the skull exposed, co-ordinates for the right (i.e. contralateral to the injury) ventral posterolateral thalamus (VPL) were calculated in relation to bregma (2.28 mm caudal, 3.2 mm lateral) (Watson and Paxinos, 2006). A small craniotomy was performed with a high-speed surgical micro-drill. The muscle overlying the lumbar vertebrae was removed, a partial laminectomy was performed to expose the L4-L6 lumbar region, and the overlying dura was removed. Once haemostasis was achieved, the surrounding muscle was coated in petroleum jelly to form a hydrophobic barrier to contain the drug. Extracellular recordings were made from VPL thalamic neurones with receptive fields on the glabrous skin of the left paw hind toes (see Fig. S1 for stereotaxically determined recording sites) using 127 μm diameter 2 MΩ parylene-coated tungsten electrodes (A-M Systems, Sequim, WA). Searching involved light tapping of the receptive field. Neurones in the VPL were classified as WDR on the basis of obtaining neuronal responses to dynamic brushing, noxious punctate mechanical (60 g) and noxious heat stimulation of the receptive field (48 °C). The receptive field was then stimulated using a wider range of natural stimuli (brush, von Frey filaments – 2, 8, 15, 26 and 60 g and heat – 35, 42, 45 and 48 °C) applied over a period of 10 s per stimulus. The heat stimulus was applied with a constant water jet onto the centre of the receptive field. Acetone and ethyl chloride (100 μl) were applied as an evaporative innocuous cooling and noxious cooling stimulus respectively (Leith et al., 2010). Evoked responses to room temperature water (25 °C) were minimal, or frequently completely absent, and subtracted from acetone and ethyl chloride evoked responses to control for any concomitant mechanical stimulation during application. Stimuli were applied starting with the lowest intensity stimulus with approximately 30–40 s between stimuli in the following order: brush, von Frey, cold, heat.

Baseline recordings were made with 25 μl vehicle (0.9% saline) applied topically to the dorsal aspect of the spinal cord after aspiration of any cerebrospinal fluid. After obtaining three baseline responses (applied in the order described with 5 min between each sets of trials, data were averaged to give control values), the vehicle was removed and 10 μg and 50 μg ondansetron hydrochloride (Claris Lifesciences, Cheshire, UK), or 50 μg and 100 μg ketanserin tartrate (Tocris, Abingdon, UK) were cumulatively applied to the spinal cord in a volume of 25 μl, and neuronal responses were characterised 20 and 40 min post-dosing; time point of peak change from baseline is plotted. The second dose was applied approximately 50–60 min after aspiration of the first dose; excess drug was washed from the cord with 25 μl vehicle (applied for 2–3 min). Drug doses were guided by previous studies (Rahman et al., 2011; Suzuki et al., 2004).

Data were captured and analysed by a CED1401 interface coupled to a computer with Spike2 v4 software (Cambridge Electronic Design, Cambridge, United Kingdom) with rate functions. The signal was amplified (x6000), bandpass filtered (low/high frequency cut-off 1.5/2 kHz) and digitised at rate of 20 kHz. Spike sorting was performed *post hoc* with Spike2 using fast Fourier transform followed by 3-dimensional principal component analysis of waveform features for multi-unit discrimination. Neurones were recorded from one site per rat; one to three neurones were characterised at each site. Stimulus evoked neuronal responses were determined by subtracting total spontaneous neuronal activity in the 10-s period immediately preceding stimulation. Spontaneous firing of individual neurones (number of spikes per second) is expressed as the mean of these 10-s periods. A total of 16 sham and 15 SNL rats were used in this study. All electrophysiological studies were non-recovery; after the last post-drug time-point, rats were terminally anesthetised with isoflurane.

### 2.4 Statistics

Statistical analyses were performed using SPSS v25 (IBM, Armonk, NY). Heat and mechanical coding of neurones were compared with a 2-way repeated measures (RM) ANOVA, followed by a Bonferroni *post hoc* test for paired comparisons. Cold, brush and spontaneous firing were compared with a 1-way repeated measures (RM) ANOVA, followed by a Bonferroni *post hoc* test for paired comparisons. Where appropriate, sphericity was tested using Mauchly's test; the Greenhouse-Geisser correction was applied if violated. Group sizes were determined by *a priori* calculations (α 0.05, 1-β 0.8). All data represent mean ± 95% confidence interval (CI). *P < 0.05, **P < 0.01, ***P < 0.001.

## 3 Results

### 3.1 Inhibition of spinal 5-HT_3_ receptors reveals tonic facilitation of noxious punctate mechanical stimulation in sham rats

**Fig. 1:**
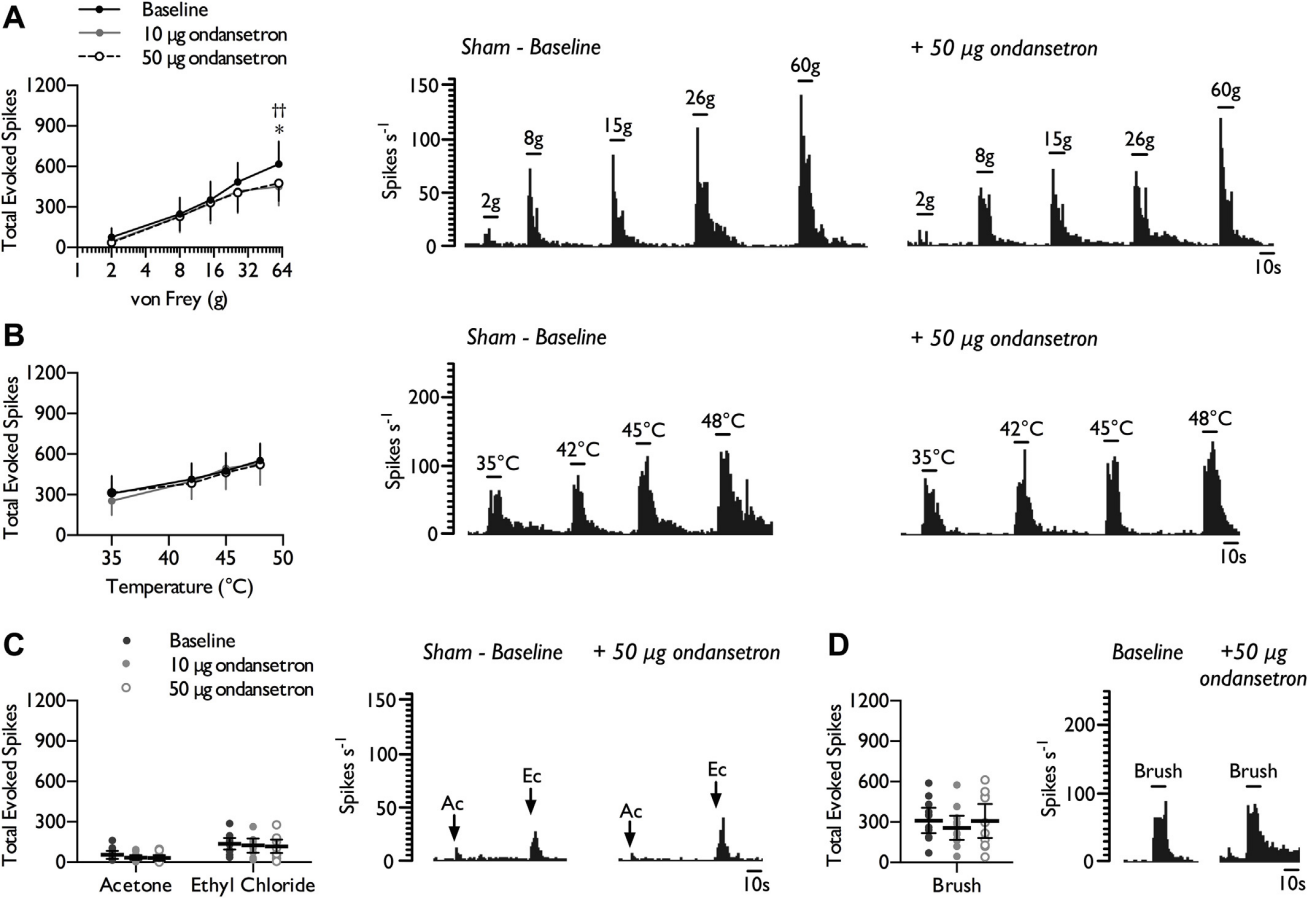
Effect of spinal 5-HT_3_ receptor inhibition on stimulus-evoked firing in the VPL of sham rats. WDR neuronal responses to punctate mechanical (*A*), heat (*B*), cold (*C*), and dynamic brush (*D*) stimuli, prior to and following spinal administration of ondansetron. Histogram traces represent typical single unit responses. Data represent mean ± 95% CI, *n* = 12 neurones from 8 rats. Asterisks (*) denote difference between baseline and 10 μg ondansetron, daggers (†) denote difference between baseline and 50 μg ondansetron, *P < 0.05, **P < 0.01. (Ac – acetone, Ec – ethyl chloride).

Once baseline responses had been obtained (summarised in Table S1), the 5-HT_3_R antagonist ondansetron (10 μg and 50 μg) was cumulatively applied to the spinal cord. At both doses, ondansetron inhibited neuronal responses to punctate mechanical stimulation selectively at the most noxious intensity of stimulation (Fig. 1*A*) (2-way RM ANOVA, *F*_2,22_ = 4.934, *P* = 0.017). The inhibitory effects of ondansetron were modality selective; no decrease in heat (Fig. 1*B*) (2-way RM ANOVA, *F*_2,22_ = 0.09, *P* = 0.915), innocuous (acetone) and noxious (ethyl chloride) evaporative cooling (Fig. 1*C*) (1-way RM ANOVA, acetone: *F*_2,22_ = 1.996, *P* = 0.16; ethyl chloride acetone: *F*_2,22_ = 0.562, *P* = 0.578), or dynamic brush evoked responses were observed (Fig. 1*D*) (1-way RM ANOVA, *F*_2,22_ = 1.958, *P* = 0.165). In addition, spinal 5-HT_3_R block did not affect overall spontaneous firing rates with only 3/12 units weakly inhibited (Baseline: 9.93 ± 8.68; 10 μg OND: 7.21 ± 6.36; 50 μg OND: 5.09 ± 4.92 spikes s^−1^) (1-way RM ANOVA, *F*_1.01,12.09_ = 1.622, *P* = 0.229).

### 3.2 Spinal 5-HT_3_ receptors mediate enhanced facilitation of punctate mechanical and noxious heat stimuli in SNL rats

**Fig. 2:**
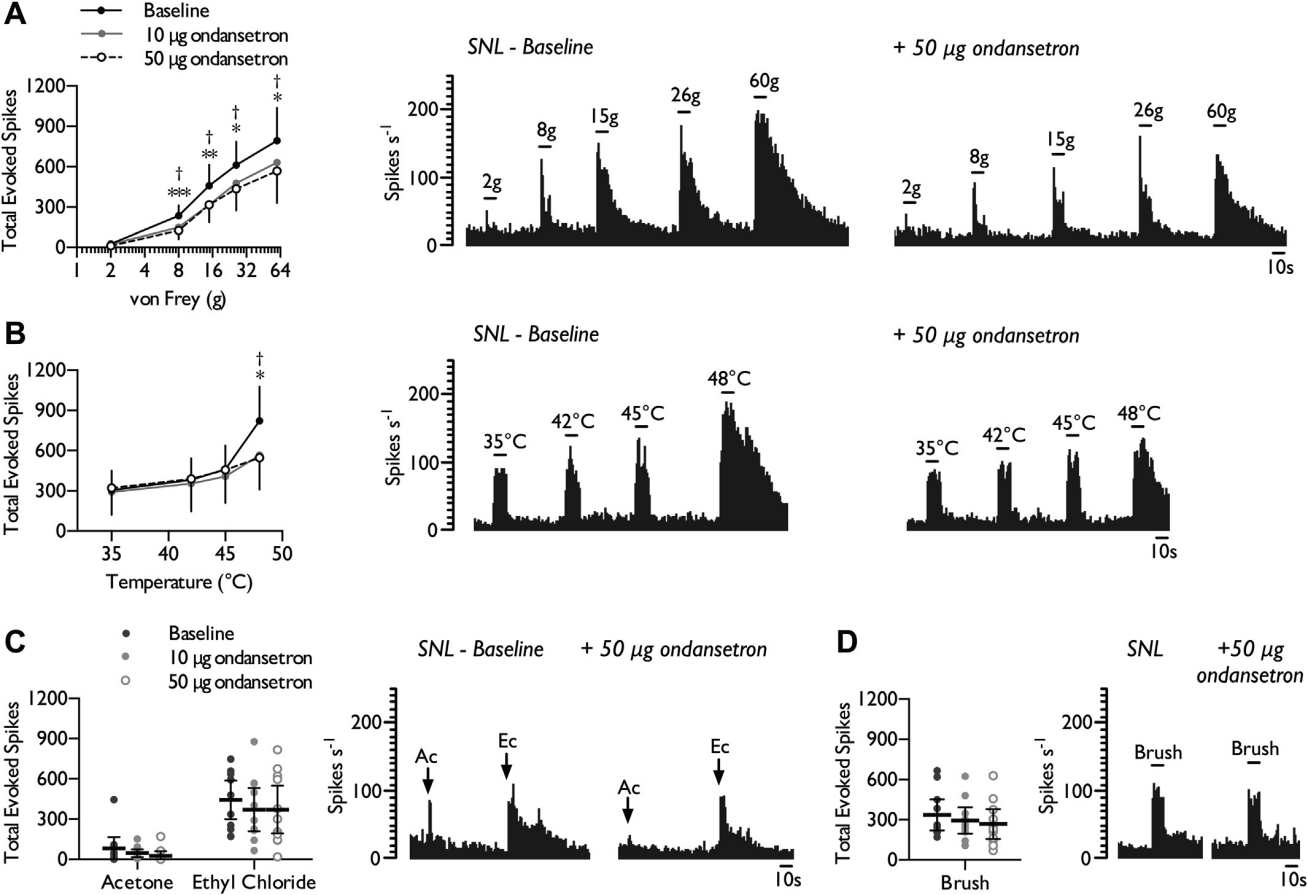
Effect of spinal 5-HT_3_ receptor inhibition on stimulus-evoked firing in the VPL of SNL rats. WDR neuronal responses to punctate mechanical (*A*), heat (*B*), cold (*C*), and dynamic brush (*D*) stimuli, prior to and following spinal administration of ondansetron. Histogram traces represent typical single unit responses. Data represent mean ± 95% CI, *n* = 11 neurones from 7 rats. Asterisks (*) denote difference between baseline and 10 μg ondansetron, daggers (†) denote difference between baseline and 50 μg ondansetron, *P < 0.05, **P < 0.01, ***P < 0.001. (Ac – acetone, Ec – ethyl chloride).

The inhibitory profile of spinal ondansetron was altered in neuropathic rats. In contrast to sham-operated rats, in SNL rats 10 μg and 50 μg ondansetron inhibited neuronal responses to lower intensity punctate mechanical stimuli in addition to the higher intensities (>15 g) that may exceed nociceptive withdrawal thresholds (Fig. 2*A*) (2-way RM ANOVA, *F*_2,20_ = 8.472, *P* = 0.0155). Furthermore, noxious heat evoked neuronal responses were now inhibited at both doses tested (Fig. 2*B*) (2-way RM ANOVA, *F*_2,20_ = 3.61, *P* = 0.046). However, there were no inhibitory effects on cooling (Fig. 2*C*) (1-way RM ANOVA, acetone: *F*_1.2,12.02_ = 3.312, *P* = 0.089; ethyl chloride acetone: *F*_2,20_ = 0.704, *P* = 0.507) or brush evoked responses (Fig. 2*D*) (1-way RM ANOVA, *F*_2,20_ = 2.547, *P* = 0.103). There was no overall effect on spontaneous activity with only 4/11 units weakly inhibited (Baseline: 14.72 ± 7.27; 10 μg OND: 11.40 ± 5.35; 50 μg OND: 10.09 ± 5.6 spikes s^−1^) (1-way RM ANOVA, *F*_1.14,11.43_ = 2.785, *P* = 0.12).

### 3.3 Inhibition of spinal 5-HT_2A_ receptors reveals minimal tonic facilitatory influence in sham rats

**Fig. 3:**
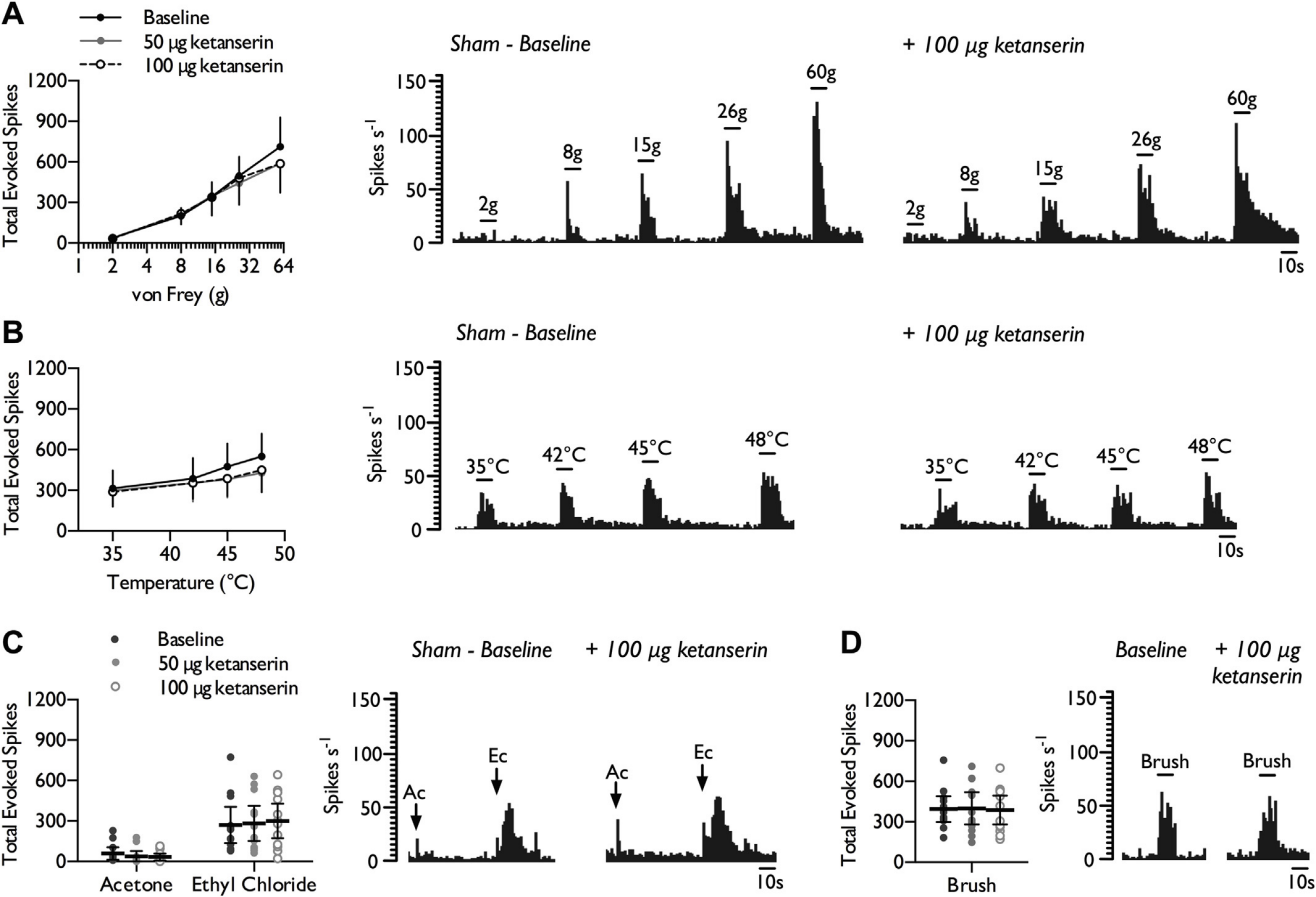
Effect of spinal 5-HT_2A_ receptor inhibition on stimulus-evoked firing in the VPL of sham rats. WDR neuronal responses to punctate mechanical (*A*), heat (*B*), cold (*C*), and dynamic brush (*D*) stimuli, prior to and following spinal administration of 50 and 100 μg ketanserin. Histogram traces represent typical single unit responses. Data represent mean ± 95% CI, *n* = 12 neurones from 8 rats. (Ac – acetone, Ec – ethyl chloride).

After sham rats were dosed spinally with the 5-HT_2A_R antagonist ketanserin (50 μg and 100 μg), we found weak evidence for tonic facilitatory activity in 4/12 units tested in the VPL. However, ketanserin had no overall effect on neuronal responses to punctate mechanical stimulation (Fig. 3*A*) (2-way RM ANOVA, *F*_2,22_ = 1.232, *P* = 0.311), heat (Fig. 3*B*) (2-way RM ANOVA, *F*_2,24_ = 3.353, *P* = 0.054), cooling (Fig. 3*C*) (1-way RM ANOVA, acetone: *F*_1.17,12.85_ = 0.801, *P* = 0.406; ethyl chloride acetone: *F*_2,22_ = 0.716, *P* = 0.5) or brush evoked responses (Fig. 3*D*) (1-way RM ANOVA, *F*_2,22_ = 0.123, *P* = 0.885). In addition, spinal ketanserin did not alter spontaneous activity in the VPL (Baseline: 8.69 ± 4.96; 50 μg KTN: 7.66 ± 4.59; 100 μg KTN: 8.70 ± 5.16 spikes s^−1^) (1-way RM ANOVA, *F*_2,22_ = 0.791, *P* = 0.466).

### 3.4 Spinal 5-HT_2A_ receptors mediate enhanced facilitation of cooling stimuli in SNL rats

**Fig. 4:**
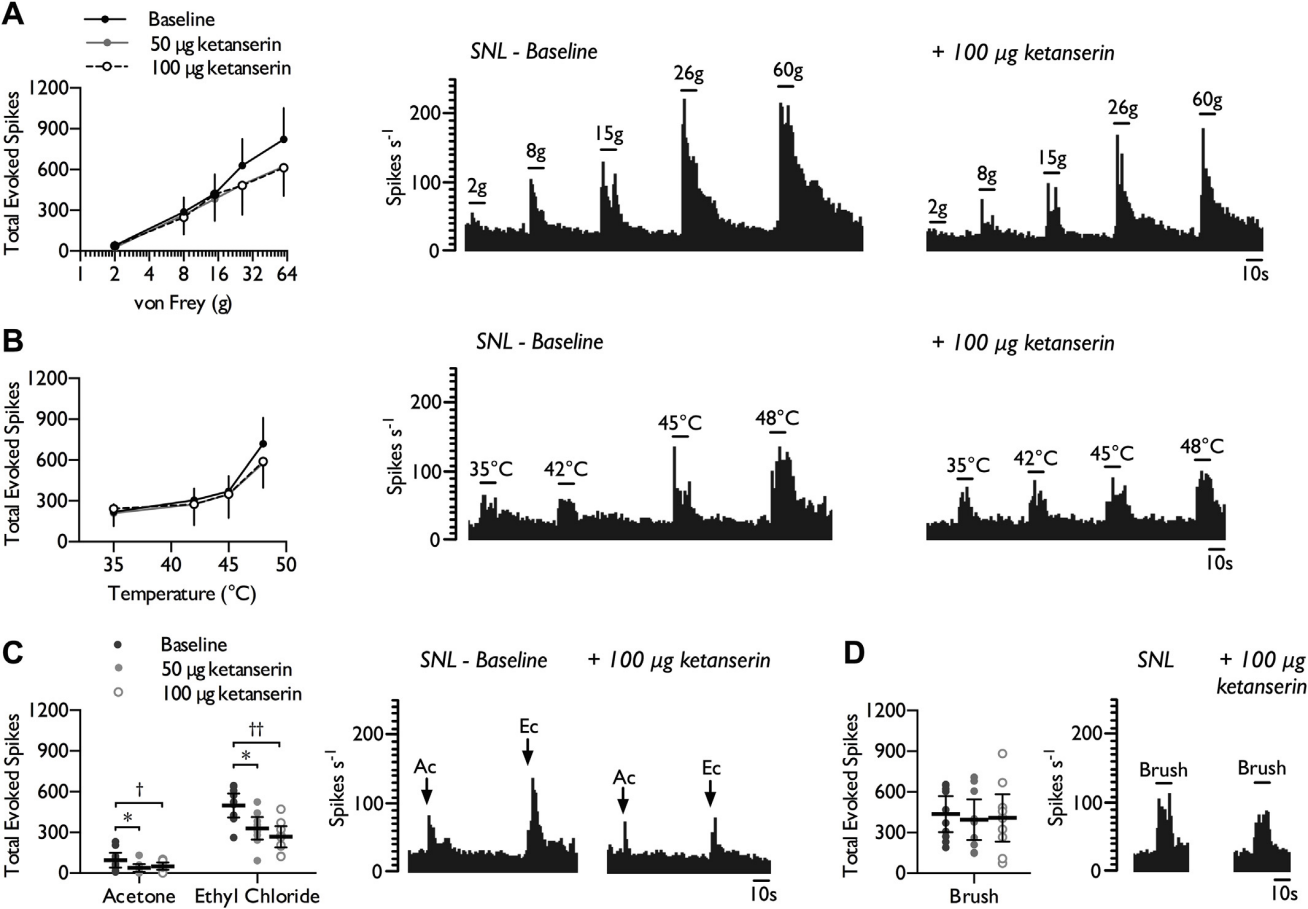
Effect of spinal 5-HT_2A_ receptor inhibition on stimulus-evoked firing in the VPL of SNL rats. WDR neuronal responses to punctate mechanical (*A*), heat (*B*), cold (*C*), and dynamic brush (*D*) stimuli, prior to and following spinal administration of ketanserin. Histogram traces represent typical single unit responses. Data represent mean ± 95% CI, *n* = 10 neurones from 8 rats. Asterisks (*) denote difference between baseline and 50 μg ketanserin, daggers (†) denote difference between baseline and 100 μg ketanserin, *P < 0.05, **P < 0.01. (Ac – acetone, Ec – ethyl chloride).

The inhibitory profile of spinal ketanserin was also altered in neuropathic rats. As observed in sham rats, there was no overall effect on punctate mechanical (Fig. 4*A*) (2-way RM ANOVA, *F*_2,18_ = 3.22, *P* = 0.064) and heat evoked responses (Fig. 4*B*) (2-way RM ANOVA, *F*_2,18_ = 1.225, *P* = 0.317). However, both 50 μg and 100 μg ketanserin inhibited neuronal responses to innocuous (acetone) and noxious (ethyl chloride) evaporative cooling (Fig. 4*C*) (1-way RM ANOVA, acetone: *F*_2,18_ = 4.595, *P* = 0.024; ethyl chloride acetone: *F*_2,18_ = 12.391, *P* = 0.00041). No inhibitory effect was observed on brush evoked responses (Fig. 4*D*) (1-way RM ANOVA, *F*_2,18_ = 0.716, *P* = 0.502), nor on spontaneous firing rates (Baseline: 18.05 ± 8.32; 50 μg KTN: 18.40 ± 9.22; 100 μg KTN: 15.63 ± 11.26 spikes s^−1^) (1-way RM ANOVA, *F*_2,18_ = 0.36, *P* = 0.703).

## 4 Discussion

In this study, we describe the intensity and modality selective tonic pro-nociceptive function of spinal 5-HT_2A_ and 5-HT_3_ receptors in the absence of nerve injury, and enhanced facilitatory roles in neuropathic conditions. Furthermore, these receptors selectively facilitated stimulus-evoked thalamic neuronal responses but not spontaneous firing in neuropathic rats. Previous studies often examined descending modulation of pain utilising spinal endpoints, either behavioural or electrophysiological. The former assay is limited to examining withdrawal threshold responses and may lack the sensitivity to decipher descending influences on spinal excitability given that modulatory medullary neurones respond selectively to intense noxious stimulation in normal conditions. Although the latter approach circumvents this shortcoming and affords the ability to examine neuronal responses to supra-threshold stimuli, the projection pathways of these neurones are rarely confirmed and effects on spontaneous neuronal firing are infrequently studied. To our knowledge, this study for the first time examines the impact of activity within bulbospinal pathways on integrative sensory processing within the VPL in normal and neuropathic states.

In normal states ON- and OFF-cells in the RVM typically exhibit ‘all-or-nothing’ responses independently of sensory modality but discriminating between innocuous and noxious inputs. In neuropathic states, ON-cells gain sensitivity to lower intensity stimuli and display exaggerated responses to noxious stimulation (Carlson et al., 2007). Correspondingly, these effects are mirrored at the spinal level following lidocaine block of the RVM (Bee and Dickenson, 2007). Spinal 5-HTRs mediate complex pro- and anti-nociceptive effects; in general, 5-HT_2A/3/4_Rs are considered to be facilitatory whereas 5-HT_1/2C/7_Rs are inhibitory (Millan, 2002). At the cellular level, 5-HT_2A_ and 5-HT_3_ receptors exert excitatory effects, the former via downstream mechanisms mediated by activation of phospholipase C, whereas the latter is ionotropic and can directly affect membrane excitability. Anatomically and functionally, both receptors are implicated in pro- and anti-nociceptive functions. A large number of GABAergic and enkephalinergic inhibitory interneurons express 5-HT_3_Rs (Huang et al., 2008), though 5-HT_2A_R localisation with GABAergic interneurones is much more limited (Wang et al., 2009). Both receptors may enhance inhibitory modulation in the superficial dorsal horn (Fukushima et al., 2009; Xie et al., 2012). However, numerous studies overwhelmingly support a net facilitatory role in acute nociceptive, inflammatory and neuropathic states (Aira et al., 2010; Bravo-Hernandez et al., 2012; Cervantes-Duran et al., 2016; Dogrul et al., 2009; Rahman et al., 2011; Suzuki et al., 2004; Tan et al., 2017; Zeitz et al., 2002). Pre-synaptically, the 5-HT_3_R is mainly expressed in myelinated neurones and low numbers of TRPV1-positive neurones (Zeitz et al., 2002), and functionally we observe facilitatory influences on mechanical but not heat evoked neuronal responses in sham rats. Although 5-HT_3_Rs are also present post-synaptically in the dorsal horn, the modality selective effects are consistent with a preferential engagement of pre-synaptic receptors by descending serotonergic brainstem projections. Neither block of spinal 5-HT_2A_ nor 5-HT_3_ receptors inhibited electrically evoked wind-up of dorsal horn neurones (Rahman et al., 2011; Suzuki et al., 2004), consistent with a pre-synaptic locus of action. The 5-HT_3_R-mediated sensitisation of TRPV1 in injured and uninjured primary afferent terminals likely leads to sensitisation to punctate mechanical and heat stimuli in neuropathic states (Kim et al., 2014). Interactions between 5-HT_3_R activity and calcium channel function may also enhance excitatory transmission (Chang et al., 2013; Suzuki et al., 2005). Neither block of 5-HT_2A_ nor 5-HT_3_ receptors altered dynamic brush-evoked neuronal responses indicating that descending facilitation is unlikely to mediate brush hypersensitivity.

We found weak evidence for tonic facilitation of noxious punctate mechanical and noxious heat evoked responses in sham rats via 5-HT_2A_Rs, broadly in line with the effects observed on spinal neuronal excitability (Rahman et al., 2011). In SNL rats, enhanced facilitation of noxious punctate mechanical and heat responses did not appear to be mediated through 5-HT_2A_Rs as effect sizes were similar to the sham group. However, spinal 5-HT_2A_R block now revealed facilitation of neuronal responses to innocuous and noxious evaporative cooling. Cold allodynia is a frequent occurrence in neuropathic conditions (Maier et al., 2010). Numerous peripheral and spinal mechanisms have been proposed to underlie this sensory disturbance (Sikandar et al., 2013a), but altered monoaminergic control should not be overlooked; noxious cold transmission is modulated by the PAG-RVM pathway in the absence of injury (Goncalves et al., 2007; Leith et al., 2010), excitability of RVM ON-cells to somatic cold stimulation is enhanced after nerve injury (Goncalves et al., 2007), and intra-RVM lidocaine reverses behavioural hypersensitivity to cooling in neuropathic rats (Taylor et al., 2007). The 5-HT_2A_R can promote excitatory transmitter release in part by inhibiting 4-amniopyridine sensitive potassium currents (Lambe and Aghajanian, 2001). In peripheral sensory neurones, transient 4-amniopyridine sensitive currents are proposed to set thresholds for cold sensitivity, and inhibition of this ‘excitatory brake’ could enhance cold transmission (Viana et al., 2002). Immunoreactivity for 5-HT_2A_Rs is largely detected in peptidergic and non-peptidergic medium-to-small sized dorsal root ganglion neurones (Okamoto et al., 2002; Van Steenwinckel et al., 2009). It does not appear that 5-HT_2A_ and 5-HT_3_ receptors are upregulated after nerve injury (Hu et al., 2016; Okamoto et al., 2002; Peters et al., 2010) but enhanced spinal gain may still arise from a combination of plasticity in descending pathways and spinal circuits (such as increased 5-HTR coupling to downstream mediators). Table 1 illustrates the balance between tonic descending inhibitory and excitatory tone in sham rats, and how this affects thalamic sensory coding across modalities and stimulus intensity. In neuropathic rats augmented serotonergic facilitation, and a concurrent loss of descending noradrenergic inhibition (Patel et al., 2018b), contributes to substantial sensory gain.

**Table 1:** Summary of descending inhibitory (DI) and facilitatory (DF) influences, mediated by spinal α_2_-adrenoceptors (Patel et al., 2018b) and 5-HT_2A/3_ receptors respectively, on VPL neuronal excitability in sham-operated and SNL rats. (+represents tonic receptor activity, +/− represents weak activity, - represents no effect of receptor block).

	Sham		SNL	
	DI	DF	DI	DF
Brush	+	–	–	–
2 g	–	–	–	–
8 g	+	–	–	+
15 g	+	–	–	+
26 g	+	–	–	+
60 g	+	+	–	+
Acetone	–	–	–	+
Ethyl chloride	+	–	–	+
35˚C	–	–	–	–
42˚C	+	–	–	–
45˚C	+	–	–	–
48˚C	+	±	–	+
Spontaneous firing	+	±	+	±

Aberrant spontaneous firing of VPL WDR neurones in SNL rats is dependent on ongoing peripheral and spinal activity (Patel et al., 2018a). Neither block of spinal 5-HT_2A_ nor 5-HT_3_ receptors inhibited spontaneous firing in sham and SNL rats. Consistent with our observations, following trigeminal nerve injury, depletion of brainstem 5-HT had no effect on the spontaneous activity of trigeminal nucleus caudalis neurones (Okubo et al., 2013). However, intrathecal ondansetron produces conditioned place preference selectively in neuropathic rats demonstrating that enhanced serotonergic activity via spinal 5-HT_3_Rs can lead to an ongoing aversive state, and supports a partial separation of sensory and affective processing (Wang et al., 2013).

Polymorphisms of the 5-HT_2A_R are associated with fibromyalgia, a condition considered to be dependent on altered central modulation (Bondy et al., 1999), and 5-HT_3_Rs are highly expressed in human dorsal root ganglion neurones (Ray et al., 2018) supporting involvement of these receptors in pain modulation in humans. Few clinical reports exist examining the effect of 5-HT_2A_ antagonists in chronic pain patients. The 5-HT_2A_R blocker sarpogrelate is not thought to be blood-brain-barrier permeable but through a peripheral mechanism may provide relief in diabetic neuropathy (Hotta et al., 1999; Ishimura et al., 1997), and after lumbar disc herniation (Kanayama et al., 2005). Likewise, the use of ondansetron clinically for analgesic purposes has been limited due to the poor penetration of the blood-brain-barrier (Simpson et al., 1992). In peripheral neuropathy patients, intravenous ondansetron had mixed effects on ongoing pain (McCleane et al., 2003; Tuveson et al., 2011), but no effect on brush allodynia (Tuveson et al., 2011), an observation that resembles the neuronal measure in this study. Similarly, in chronic back pain patients tropisetron, a highly selective 5-HT_3_R antagonist, had no overall effect on the intensity of ongoing pain and minimal effects on secondary measures of sensitisation (Neziri et al., 2012). Although not considered a neuropathic condition, fibromyalgia is idiopathic and is characterised by widespread sensitisation and musculoskeletal pain, but also disturbances in descending modulation (O'Brien et al., 2018). Tropisetron was effective in a subgroup of these patients reducing the number of painful pressure points and associated pain intensity scores (Farber et al., 2000), and again bears a marked resemblance to the neuronal measures described here and previously (Suzuki et al., 2004). Of course, a caveat of these studies is the systemic route of dosing and the inability to rule out involvement of peripheral mechanisms or other centrally mediated processes. However, the concordance between the psychophysical measures in fibromyalgia patients with systemic treatment, and the thalamic neuronal measures following spinal dosing implies similar underlying processes are targeted.

The modality and intensity dependent facilitatory role the 5-HT_3_R supports the notion that 5-HT_3_R antagonists are more effective for alleviating static/punctate mechanical hyperalgesia, and could merit further clinical investigation in patients stratified according to these sensory disturbances. Several have advocated a move to mechanism-based treatment selection and that sensory profiles of patients represent surrogate measures for underlying mechanisms (Baron et al., 2017; Bouhassira and Attal, 2016; Max, 1990; Woolf et al., 1998). From a clinical presentation, determining enhanced descending facilitation as a mechanism present in a patient poses some difficulties. Conditioned pain modulation (CPM), an assay through which a heterotopic conditioning stimulus inhibits the perceived intensity of a test stimulus, provides a readout of the integrity of endogenous pain modulation. CPM is frequently diminished in chronic pain patients (Vaegter and Graven-Nielsen, 2016; Yarnitsky et al., 2012), but this net loss of inhibition might result from decreased noradrenergic inhibitory tone, increased facilitatory drive, or a combination of both. Baron and colleagues describe three sensory phenotypes (‘mechanical’, ‘thermal’ and ‘sensory loss’) in neuropathic patients (Baron et al., 2017), though it is unclear whether inefficient CPM correlates with any of these. The sensory profile of the SNL model shares features with the ‘mechanical’ and ‘thermal’ phenotypes (Dickenson and Patel, 2018; Patel et al., 2018a), and diffuse noxious inhibitory controls are absent in this model (Bannister et al., 2015). Speculatively, based on the modality and intensity dependent roles, enhanced descending facilitation terminating on 5-HT_2A_ and 5-HT_3_ receptors may be associated with sub-groups within the ‘mechanical’ and ‘thermal’ sensory phenotypes; our current observations could help shape translational pharmacological studies.

## Author contributions

RP and AHD, conception and design of study; RP, performed experiments; RP, analysed data; RP and AHD, interpreted results of experiments; RP, prepared figures; RP, drafted manuscript; RP and AHD, edited and revised manuscript. Both authors approved the final manuscript.

## Funding sources

This study was funded by the Wellcome Trust Pain Consortium [102645 – Defining pain circuitry in health and disease].

## Conflicts of interest

None to declare.
