# Supplementary material for: Modality selective roles of pro-nociceptive spinal 5-HT2A and 5-HT3 receptors in normal and neuropathic states
Source: Neuropharmacology. 2018 Dec;143:29–37. doi: 10.1016/j.neuropharm.2018.09.028 (PMC6277848; doi:10.1016/j.neuropharm.2018.09.028)
Supplement: Figure S1 [file mmc1.docx]

**
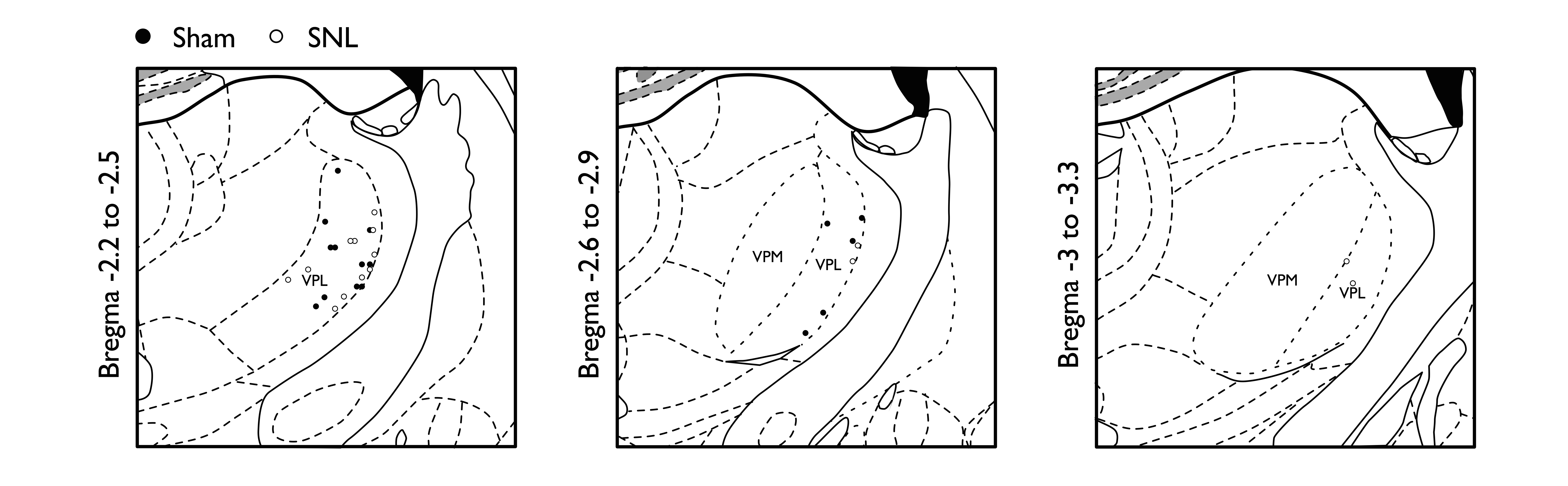
**

**Figure S1.** Recording sites within the ventral posterolateral thalamus from 16 sham and 15 neuropathic rats. Filled circles (●) represent sham, open circles (○) represent spinal nerve ligated (SNL) rat experiments.
