# Supplementary material for: Modality selective roles of pro-nociceptive spinal 5-HT2A and 5-HT3 receptors in normal and neuropathic states
Source: Neuropharmacology. 2018 Dec;143:29–37. doi: 10.1016/j.neuropharm.2018.09.028 (PMC6277848; doi:10.1016/j.neuropharm.2018.09.028)
Supplement: Table S1 [file mmc2.docx]

|  | **Sham *n*=24** | | | **SNL *n*=21** | | |
| --- | --- | --- | --- | --- | --- | --- |
|  |  |  |  |  |  |  |
| **Brush** | 353 | ± | 30.40 | 384 | ± | 38.80 |
| **2g** | 57 | ± | 15.32 | 34 | ± | 7.13 |
| **8g** | 224 | ± | 29.76 | 260 | ± | 28.37 |
| **15g** | 347 | ± | 37.57 | 442 | ± | 45.59 |
| **26g** | 491 | ± | 43.62 | 620 | ± | 55.62 |
| **60g** | 665 | ± | 60.62 | 808 | ± | 72.10 |
|  |  |  |  |  |  |  |
| **35°C** | 313 | ± | 34.54 | 266 | ± | 36.17 |
| **42°C** | 402 | ± | 41.85 | 345 | ± | 40.93 |
| **45°C** | 476 | ± | 46.87 | 418 | ± | 46.91 |
| **48°C** | 551 | ± | 115.46 | 773 | ± | 70.39 |
|  |  |  |  |  |  |  |
| **Acetone** | 57 | ± | 11.95 | 88 | ± | 21.90 |
| **Ethyl chloride** | 203 | ± | 33.86 | 470 | ± | 37.69 |
|  |  |  |  |  |  |  |
| **Spontaneous firing (spikes/s)** | 9.36 | ± | 2.23 | 16.30 | ± | 2.32 |

**Table S1.** Baseline characterisations of wide dynamic range neurones in the ventral posterolateral thalamus of sham and spinal nerve ligated rats. Unless otherwise specified, data represent mean total evoked spikes ± 95% CI.
